# A Rare Case Report of Thyroglossal Duct Cyst Carcinoma Coexisting with Thyroid Carcinoma in an Adolescent

**DOI:** 10.1155/2023/6640087

**Published:** 2023-09-05

**Authors:** Kleanthi Mylopotamitaki, Dionisios Klonaris, Georgios Kazamias, Christos Simandirakis, Irene Vourliotaki, Efthimios Karakostas

**Affiliations:** ^1^Department of Otorhinolaryngology-Head and Neck Surgery, General Hospital of Heraklion “Venizeleio-Pananeio”, Heraklion, Crete, Greece; ^2^University of Crete, School of Medicine, Greece; ^3^Department of Pathology, General Hospital of Heraklion “Venizeleio-Pananeio”, Heraklion, Crete, Greece; ^4^Department of Endocrinology, General Hospital of Heraklion “Venizeleio-Pananeio”, Heraklion, Crete, Greece

## Abstract

**Background:**

Thyroglossal duct cysts (TDC) represent approximately 70% of all congenital neck masses, and up to 1% of them contain thyroid tissue malignancies. Clinical presentation of TDC carcinomas is usually indistinguishable from benign tumors preoperatively, and differential diagnosis can be challenging. We present a rare case of TDC carcinoma concurrent with thyroid cancer in an adolescent. *Case Presentation*. A 16-year-old Caucasian female, otherwise healthy, was referred with a painless, gradually expanding lump on the neck. Physical examination revealed a well-circumscribed, moderately hard, tender mass of the anterior neck midline anteroinferior to the hyoid bone. Imaging findings suggested TDC as the most likely diagnosis. The patient had a Sistrunk procedure under general anesthesia. Histopathological findings diagnosed a BRAF^V600E^-positive papillary thyroid carcinoma (PTC) in a TDC. A thyroid gland and neck ultrasound revealed a highly suspicious finding for malignancy right level VI lymph node, which was not confirmed by fine needle aspiration cytology (FNAC). Under general anesthesia, total thyroidectomy and central compartment lymph node neck dissection were performed. Histopathological findings revealed a thyroid parenchymal locus of PTC, as well as three lymph nodes infiltrated by PTC. The patient received adjuvant radioactive iodine ablation (RAI) therapy and is closely followed.

**Conclusion:**

TDC carcinomas in conjunction with thyroid carcinomas in young patients are rare. Preoperative diagnosis can be challenging, as the vast majority of neck masses in young patients are benign in nature, and most malignant tumors lack specific clinical features. The diagnostic accuracy of FNAC is considered unsatisfactory due to its frequently cystic nature. Definitive diagnosis is based on histopathological findings. Clinicians should maintain a high level of suspicion for coexisting thyroid malignancies. Although surgical extirpation of the malignancy is considered standard of care, the treatment of TDC cancer should always be individualized by a multidisciplinary team.

## 1. Introduction

Thyroglossal duct cysts (TDC) represent 70% of all congenital neck masses, with a prevalence of 7% in the general population [1]. They arise from remnants of the thyroglossal duct that failed to regress during gestation. They grow along the embryologic descending route of the thyroid gland, starting at the foramen cecum and ending at the level of the cricoid cartilage and upper trachea [2]. Sixty percent of these masses are present at the level of the hyoid bone, while 24% are suprahyoid and 13% are infrahyoid [3, 4]. Most of these cysts have an average size of 2 to 4 cm [5]. Ectopic thyroid tissue may be present in up to 50% of these, which can be explained embryologically [3, 6]. The vast majority of TDCs are benign; however, up to 1% may contain malignant neoplasms [7]. Most of these cancers are variants of papillary thyroid carcinoma (PTC), with a median age of initial diagnosis at 40 years [8–10]. In 20 to 60% of cases, they coexist with a thyroid gland PTC [11, 12]. Therefore, a diagnostic dilemma exists as to whether a TDC carcinoma is a primary tumor or metastatic from the thyroid gland.

Identifying a TDC carcinoma is generally indistinguishable from benign tumors preoperatively [8, 13]. Currently, there is no established consensus on TDC malignancy treatment, especially in adolescents [14]. The universal recommendation for these patients is the Sistrunk procedure, performed alone in low-risk patients, and only high-risk patients receive additional treatments [3, 15]. Further surgical management and adjuvant therapy after primary surgery are contemporary debates among clinicians due to the lack of data from the literature. [14]. We present a case of PTC of a DTC coexisting with PTC of the thyroid gland in an adolescent.

## 2. Case Report

**Figure 1:**
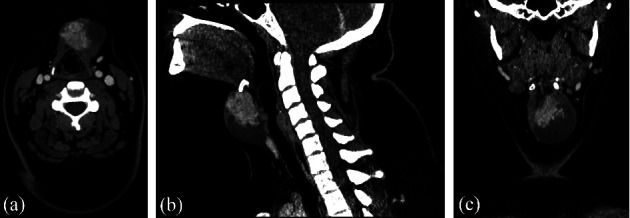
Contrast-enhanced CT showing a sizeable (4.38 × 4.26 cm) well-circumscribed heterogeneous mass anteroinferior to the hyoid bone in axial (a), sagittal (b), and coronal (c) planes.

A 16-year-old Caucasian female, otherwise healthy, was referred to our institution with a painless, gradually expanding lump on her neck. ENT examination revealed a mass located in the neck midline, anteroinferior to the hyoid bone. The mass was well-circumscribed, moderately hard, fixed, and tender at palpation and followed swallowing movements. No other significant findings were found on the rest head and neck examination, including flexible fiberoptic upper airway endoscopy. Thyroid function tests were also normal. Contrast-enhanced computerized tomography (CT) revealed a sizeable (4.38 × 4.26 cm) well-circumscribed mass anteroinferior to the hyoid bone, showing a moderate heterogeneous contrast enhancement (Figure 1). The imaging findings suggested that the most likely diagnosis was TDC. Differential diagnoses included other congenital benign masses of mixed composition or a neck tumor of mesenchymal origin.

**Figure 2:**
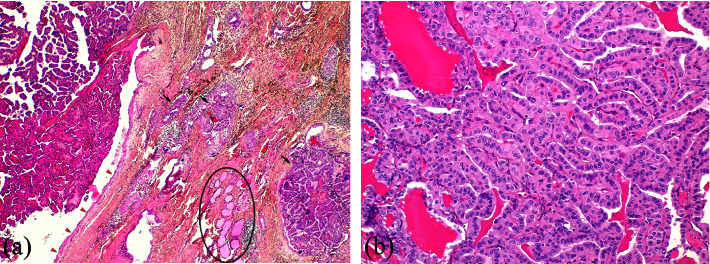
(a) Papillary thyroid carcinoma arising in a thyroglossal duct cyst (left) exhibits classical papillary morphology with invasion of the cyst wall (indicated by black arrows). The cyst is lined by stratified squamous epithelium (indicated by red arrowheads), and normal thyroid follicles are present in the cyst wall (indicated by a black circle) (H&E stain, 40x magnification). (b) The carcinoma exhibits tall cell features in approximately 20% of the tumor (H&E stain, 200x magnification).

**Figure 3:**
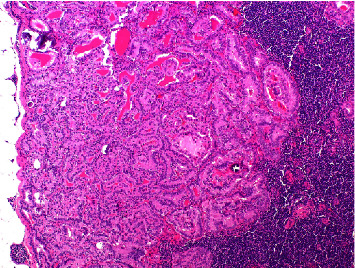
The presence of metastatic papillary carcinoma with tall cell features was observed in a lymph node at level VI (H&E stain, 100x magnification).

The patient had a Sistrunk procedure under general anesthesia. Intraoperatively, the mass was found adherent to the hyoid bone, and the middle portion of the latter was excised along with the entire thyroglossal duct tract. The patient had an uneventful postoperative course. Permanent histopathology revealed a BRAF^V600E^-positive classic PTC in a TDC (Figure 2(a)), exhibiting tall cell features in approximately 20% of the tumor cells (Figure 2(b)). According to the latest WHO classification, the diagnosis of the tall cell subtype of PTC requires at least 30% of tumor cells to display tall cell features [16]. A thyroid gland and neck ultrasound revealed highly suspicious findings for malignancy right level VI lymph node of 8 mm in size with increased echogenicity. Fine needle aspiration cytology (FNAC) of the lymph node revealed no malignant elements, and FNAC thyroglobulin lavage (FNAC-Tg) was negative. Under general anesthesia, total thyroidectomy and central compartment lymph node neck dissection were performed with no postoperative complications. Histopathological findings revealed a 6 mm parenchymal locus of PTC in the left thyroid lobe. Furthermore, three lymph nodes were infiltrated by PTC, none larger than 6 mm and without extranodal extension (Figure 3). The patient received 120 mCi of radioactive iodine (^131^I) (RAI) ablation therapy as adjuvant treatment and is now closely followed. One year postoperatively, there is no clinical or radiological evidence of disease recurrence.

## 3. Discussion

Brentano first described the rare entity of TDC carcinoma in 1911 [17]. Since then, approximately 300 cases have been reported worldwide [18]. Especially in the pediatric population, this clinical entity is very rare, with approximately 30 previously reported cases [19]. Up to 80% of TDC carcinomas are of PTC type [20]. The differential diagnosis includes a PTC that arises from the rostral end of the pyramidal lobe [21]. Most cases of TDC carcinoma are diagnosed in the fourth decade of life, and females are affected slightly more often than males in a 3 : 2 ratio [9, 10].

Some authors have proposed several high-risk characteristics of TDC carcinoma, such as age over 45 years, maximum tumor diameter greater than 1.5 cm, previous radiation exposure, nodal disease, cyst wall invasion, positive histopathological margins, and a tumor in the thyroid gland on radiological evaluation [22]. The BRAF^V600E^ mutation has also been associated with aggressive clinical and pathological features [23]. Regarding metastatic disease, regional lymph node metastases of TDC carcinoma occur at a low rate, observed in only 7.7% of reported cases [3]. Local invasion also rarely occurs, and the risk of metastasis is less than 2% [21]. Recent reports show a favorable prognosis, with survival rates reported of 99.4–100% and 95.6% at 5 and 10 years, respectively [4, 22].

There is a strong debate about the origin of TDC carcinomas. The etiology is explained by either de novo theory or metastatic theory [13]. Currently, most authors support the de novo theory, which is based on the carcinogenesis of ectopic thyroid tissue within the cyst and the absence of metastatic disease [3, 24, 25]. Metastatic theory suggests the existence of metastatic disease due to an occult primary thyroid lesion [14]. Furthermore, the diagnosis of primary carcinoma of the thyroglossal duct is one of exclusion using the Widström criteria [26].

The clinical presentation and characteristics of a TDC carcinoma are generally indistinguishable from benign TDCs [8, 13], making the preoperative diagnosis challenging [8, 27]. A detailed clinical history is essential for an accurate diagnosis before surgery [27]. The findings indicative of malignancy increased the growth rate and the presence of a palpable firm mass [28]. While the initial imaging modality of choice is ultrasound [29], CT and MRI play a critical role in preoperative diagnosis, staging of malignancy, and therapeutic approach [28]. Imaging findings can reveal a solid mass with invasive features indicative of malignancy, but a definitive diagnosis cannot be made [27, 28]. There is debate over the utility of FNAC or core needle biopsy preoperatively [4]. FNAC is a safe, well-tolerated, and cost-effective procedure for diagnosing thyroglossal duct lesions [30]. However, the sensitivity of FNAC is relatively low due to the cystic nature of the lesion [31]. A definitive diagnosis with FNAC is achieved in about 53–60% of cases [32, 33]. Therefore, FNAC is recommended when characteristics suspicious for malignancy are present, such as calcifications on ultrasound [11]. In a recent study, Rayess et al. reported that 73.3% of TDC carcinomas were diagnosed as incidental findings in the final histopathology analysis and another 20.6% preoperatively based on the FNAC analysis [4].

Regarding therapeutic strategies, there is no consensus on specific surgical management for a malignant disease that arises from a TDC [14]. The universal recommendation is the Sistrunk procedure, which involves complete excision of the thyroglossal duct tract, as well as the central portion of the hyoid bone [15]. The Sistrunk procedure is performed alone in patients with low-risk tumors and normal thyroid gland ultrasounds, whereas only high-risk patients undergo additional treatments [3]. Furthermore, surgical management and adjuvant therapy, such as RAI, are controversial among clinicians [14]. In the event of a preoperatively diagnosed TDC carcinoma, some authors recommend routinely performing a total thyroidectomy as well, even in cases with normal thyroid gland diagnostic workup [7, 34]. However, total thyroidectomy is not universally accepted as the standard of care for TDC carcinoma, as thyroid gland involvement is present only in 33–45% of cases [18]. In summary, total thyroidectomy and neck dissection should be considered in high-risk patients with concurrent thyroid malignancy or nodal disease [35]. Routine elective neck dissection is not indicated for these patients in the absence of clinically palpable nodes or suspicious findings on ultrasound or CT, due to the low risk of metastatic spread to lymph nodes [14]; it should be reserved for clinically positive nodal disease [14, 18]. Furthermore, there is also no clear recommendation on the indications and timing of RAI treatment [14]. However, it is recommended to consider RAI treatment in patients with large tumors and nodal disease or in patients with malignancy in both the thyroid gland and the TDC [14].

## 4. Conclusion

Reports in the literature of TDC carcinoma coexisting with thyroid gland PTC in young patients are rare. Preoperative diagnosis is challenging due to the fact that the vast majority of congenital neck masses in adolescents are benign in origin, and most cancers lack specific clinical characteristics. The use of FNAC is controversial due to the cystic nature of the lesion. In most cases, the final histopathological findings determine the presence of carcinoma in a TDC. Clinicians should maintain a high level of suspicion for coexisting thyroid malignancies when treating these patients. Complete disease extirpation via a Sistrunk procedure is vital for a favorable outcome. TDC cancer should be further managed according to individualized risk stratification by a multidisciplinary team.

## Data Availability

All necessary data supporting the study findings are incorporated in the article.
